# Icariin-Releasing 3-Dimensionally Printed Scaffolds for Alveolar Cleft Reconstruction

**DOI:** 10.34133/bmr.0199

**Published:** 2025-05-30

**Authors:** Soomin Park, Alexander B. Pascal, Sidney B. Eisig, Meng Feng, Hun Jin Jeong, Elen Zhu, Emily Zhang, Chang Hun Lee

**Affiliations:** ^1^ Center for Dental and Craniofacial Research, College of Dental Medicine, Columbia University Medical Center, New York, NY 10032, USA.; ^2^ Section of Hospital Dentistry/Division of Oral and Maxillofacial Surgery, College of Dental Medicine, Columbia University Medical Center, New York, NY 10032, USA.; ^3^ Veterinary Operations, Institute of Comparative Medicine (ICM), Columbia University Medical Center, New York, NY 10032, USA.

## Abstract

Each year, 1 in every 700 babies is born with an orofacial cleft in the USA. Despite a well-established protocol for early cleft repair, the alveolar cleft persists during craniofacial growth. Current surgical treatments with bone grafts for alveolar cleft often provide inadequate nasal base support and insufficient alveolar bone volume for permanent tooth eruption. Here, we developed 3-dimensionally printed polycaprolactone scaffolds with controlled delivery of icariin (ICA) to facilitate bone reconstruction. After establishing a reliable fabrication process, we determined the optimal loading dose and release kinetics of ICA for induced osteogenic differentiation of bone marrow mesenchymal stem/progenitor cells and mineralized tissue formation in vitro. Then, the ICA-releasing polycaprolactone scaffolds with the preoptimized dose were implanted into rats with full-thickness maxillary defects. Up to 8 weeks, micro-computed tomography analyses demonstrated significantly accelerated bone healing and defect closure with an ICA-releasing scaffold compared to scaffold alone and defect controls. Histology consistently confirmed the formation of dense woven bone with ICA-releasing scaffolds in contrast to unclosed gaps and soft tissue infiltration in controls. Our findings suggest the significant potential of ICA-releasing 3-dimensionally printed scaffolds to serve as a patient-focused and custom-built bone graft to improve the clinical outcome of alveolar cleft reconstruction.

## Introduction

**Fig. 1.**
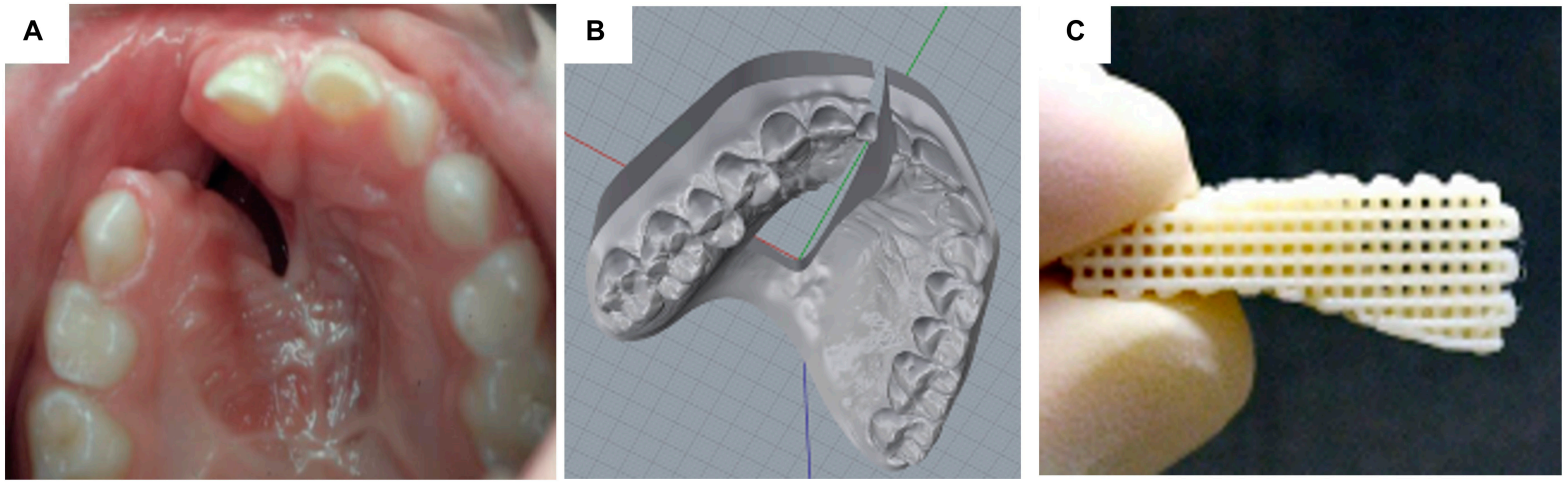
The potential of 3-dimensionally printed (3D-printed) scaffolds for repairing alveolar and maxillary clefts. The anatomical shape and dimension of the patient-specific defect (A) can be readily modeled in computer-aided design (CAD) (B) to construct ready-to-implant biodegradable scaffolds (C).

One in every 700 babies is born with orofacial cleft in the USA each year, caused by the failure or partial fusion of the appropriate hard and/or soft tissues [1]. Disruption during an early stage of embryogenesis between 4 and 12 weeks results in 2 unfused palatal shelves [2]. Untreated children with cleft lip and palate may develop speech, hearing, nutrition, and social developmental disorders [2]. On average, a patient with cleft lip and palate has 148.5 healthcare interactions, with 10.5 surgical procedures between the ages of 0 and 18 years old [3]. The median cost of care for the cleft lip and palate is estimated at US$73,398 per patient [3]. The lip and palate are usually repaired by 12 months of age. However, there is frequently a residual alveolar cleft with oral–nasal communication (Fig. 1A). Alveolar cleft osteoplasty is an essential and time-sensitive aspect of comprehensive treatment where a successful reconstruction must close the remaining oronasal fistula, establish the nasal skeletal base, and facilitate the eruption and maintenance of teeth in the cleft area [4]. The gold standard for a bone graft is by harvesting iliac crest autograft, which could result in acute or chronic graft disturbances postsurgically [5]. Commonly used biologics like recombinant human bone morphogenetic protein 2 (rhBMP-2), often combined with a demineralized bone matrix for structural support, are not Food and Drug Administration approved for use in children under 18, who are the typical target population for alveolar cleft osteoplasty [5]. Complications associated with rhBMP-2 include inflammation, excessive bone proliferation, and ectopic bone formation [6]. In addition, the high cost of rhBMP-2 may increase the cost of care [7], and rhBMP-2 may not provide adequate bulk to the grafted alveolus.

To overcome the limitations, tissue engineering approaches using 3-dimensional (3D) scaffolds, bioactive cues, and cells have been applied to facilitate alveolar cleft reconstruction [8,9]. In particular, 3D printing has received attention as a versatile tool for fabricating a customized scaffold based on a computer-aided design model of a patient-specific defect site (Fig. 1B and C). A wide range of applicable materials and a readable control of internal microstructure are among the unique advantages of 3D printing to construct scaffolds as cleft grafts [10]. Given the benefits, various 3-dimensionally printed (3D-printed) scaffolds seeded with osteogenic cells have been tested for alveolar bone reconstruction in animal models [10,11]. Despite the promising research progress, there is no well-established bone graft or biomaterial that reliably regenerates moderate to large complex alveolar bone in cleft patients without complications. Moreover, cell transplantation with scaffolds may suffer from translational barriers associated with its regulatory pathway [12,13].

Accordingly, there is an emerging research interest in endogenous tissue engineering through cost-effective alternatives to biologics that can minimize the need for autologous grafting. This study explored the potential of the 3D-printed scaffold with controlled delivery of icariin (ICA) to facilitate alveolar bone healing with cleft reconstruction surgery. ICA is a flavonoid derived from *Epimedium* (Berberidaceae). ICA has been found to improve immunity and the cardiovascular system, promote antioxidation, osteogenic differentiation of bone marrow mesenchymal stem cells (MSCs), and migration of MSCs through the mitogen-activated protein kinase pathway [14,15]. Our previous work with ICA has shown that it promotes osteogenic differentiation of stem/progenitor cells [16]. Other authors have demonstrated increased osteogenic differentiation, reduced pro-inflammatory cytokines, and suppressed osteoclastic activities [17]. Sun et al. [18] tested the efficacy of controlled release of ICA molecules in calcium phosphate scaffolds, promoting the healing of critical bone defects in beagle skulls. Liu et al. [19] reported that a slow-release ICA from siliceous mesostructured scaffolds seeded with MSCs promoted bone formation in critical-size bone defects in rats, with significant upregulation of osteogenesis- and angiogenesis-related genes and proteins, such as Runx2, alkaline phosphatase, osteocalcin, and vascular endothelial growth factors.

In this study, we established an effective and reliable protocol for delivering ICA in 3D-printed polycaprolactone (PCL) scaffolds and determined the optimal dose and release kinetics to promote osteogenic differentiation of MSCs. Then, we investigated the in vivo efficacy of the ICA-releasing PCL scaffolds for cleft bone regeneration in a rat model. Our findings suggest the notable efficacy of ICA-releasing 3D-printed scaffolds in promoting alveolar bone healing and osteointegration, with the potential to reduce the number of cleft reconstructive surgeries.

## Methods

### Preparation of ICA-loaded PCL

**Fig. 2.**
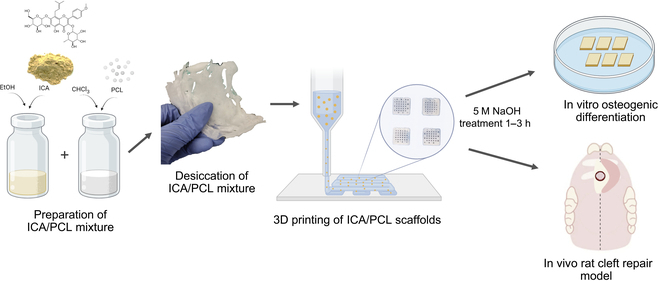
Fabrication process of an icariin (ICA)-loaded polycaprolactone (PCL) scaffold for in vitro and in vivo studies. The ICA/PCL mixture is prepared and desiccated, followed by breaking into pellets. After being loaded in a high-temperature cartridge, ICA/PCL was 3D-printed into 3D scaffolds. After surface treatment with 5 M NaOH and sterilization, the prepared scaffolds were used for in vitro osteogenic tests and in vivo studies for cleft repair. EtOH, ethanol.

Given the dose-dependent effects and cytotoxicity of ICA released from different delivery vehicles [16,18–20], we prepared 3 ICA doses (0.1, 0.3, and 0.6 wt%) loaded in PCL. As summarized in Fig. 2, PCL (50,000 Mw, Polyscience Inc., Warrington, PA, USA) was dissolved in chloroform at 2.29 g/6 ml to achieve a viscous mixture of PCL in a 10-ml glass vial. Then, a mixture of 50 mg of ICA and 1 ml of 100% ethanol (EtOH) was prepared separately as a stock solution. To prepare an ICA/PCL mixture of 0.1 wt% ICA/PCL, 45.8 μl of the 50 mg/ml ICA/EtOH mixture was thoroughly mixed into the glass vial that had a PCL and chloroform mixture. The prepared material was poured into a glass petri dish and left to dry for 24 h as a thin layer under a ventilator. To achieve 0.3 wt% ICA, 137.5 μl of the 50 mg/ml ICA/EtOH mixture was mixed with the PCL/chloroform mixture to achieve 0.3 wt% ICA/PCL. For 0.6 wt% ICA, 274.8 μl of the 50 mg/ml ICA/EtOH mixture was combined with the PCL/chloroform mixture. The control material was prepared with the PCL/chloroform mixture only. After complete evaporation of chloroform and EtOH, each material was sliced and obtained as small pellets.

### Fabrication of 3D-printed ICA-loaded PCL scaffolds

Scaffolds loaded with ICA at different concentrations were fabricated using 3D-Bioplotter (EnvisionTec, Germany). Briefly, 0.1, 0.3, or 0.6 wt% ICA/PCL or PCL-only pellets were loaded into high-temperature printer cartridges. The cartridges were then heated up to 120 °C, and ICA-embedded PCL microfibers were dispensed through a microneedle to construct 3D scaffolds (5 mm × 5 mm × 1 mm, length × width × height) with repeats of 400-μm microstrands and 200-μm interstrand channels, confirmed by microscopic measurements per our previous studies [21,22]. The 3D-printed scaffolds were then treated with 5 M NaOH for 1 to 3 h to create surface porosity, facilitating release per our previous studies [23]. After air-drying for 24 h, the scaffolds were sterilized by ethylene oxide for 24 h and ventilated at room temperature for a week.

### In vitro release kinetics of ICA

The 3D-printed ICA/PCL scaffolds were incubated in phosphate-buffered saline at 37 °C for up to 28 d with gentle agitation. The released amount of ICA in the supernatants collected at selected time points (*n* = 3 per sample; 5 samples per group) was measured by an ultraviolet spectrophotometer (Lambda 800, PerkinElmer, USA) at 270 nm following the established protocols from previous studies [24,25]. The ICA concentrations were quantified using a standard curve of ICA from 0.1 to 10 mM prepared in Dulbecco’s phosphate-buffered saline.

### Mechanical properties

The compressive properties of scaffolds (5 mm × 5 mm × 1 mm, pore size 200 μm) were measured by a UniVert testing system (CellScale, Waterloo, ON) under a constant displacement of 1 mm/min. The compressive moduli of the scaffolds were calculated from the stress–strain curves.

### Osteogenic differentiation of MSCs in ICA/PCL scaffolds

MSCs from human bone marrow (AllCells, Alameda, CA) were seeded into the scaffolds by infusing cell-suspended type I collagen gel (1 × 10^6^ cells/ml) into the microchannels of the scaffolds followed by 1-h incubation at 37 °C for gel formation, per our prior works [13,23]. The MSC-seeded scaffolds were cultured in osteogenic induction media; low-glucose Dulbecco’s modified Eagle medium was supplemented with 100 nM dexamethasone, 10 mM β-glycerophosphate, and 0.05 mM ascorbic acid-2-phosphate. After 4 weeks of culture, all constructs were harvested for histological analysis with hematoxylin and eosin, Masson trichrome, and Alizarin Red (AR) staining. AR staining was quantified by a colorimetric analysis using ImageJ (*n* = 15 per group). Quantitative reverse transcription polymerase chain reaction was performed to evaluate the expressions of osteogenic genes, including COL1A1, integrin-binding sialoprotein (IBSP), and bone gamma-carboxyglutamate protein (BGLAP) using TaqMan assay per our well-established methods [13,23].

### In vivo alveolar cleft bone reconstruction model

**Fig. 3.**
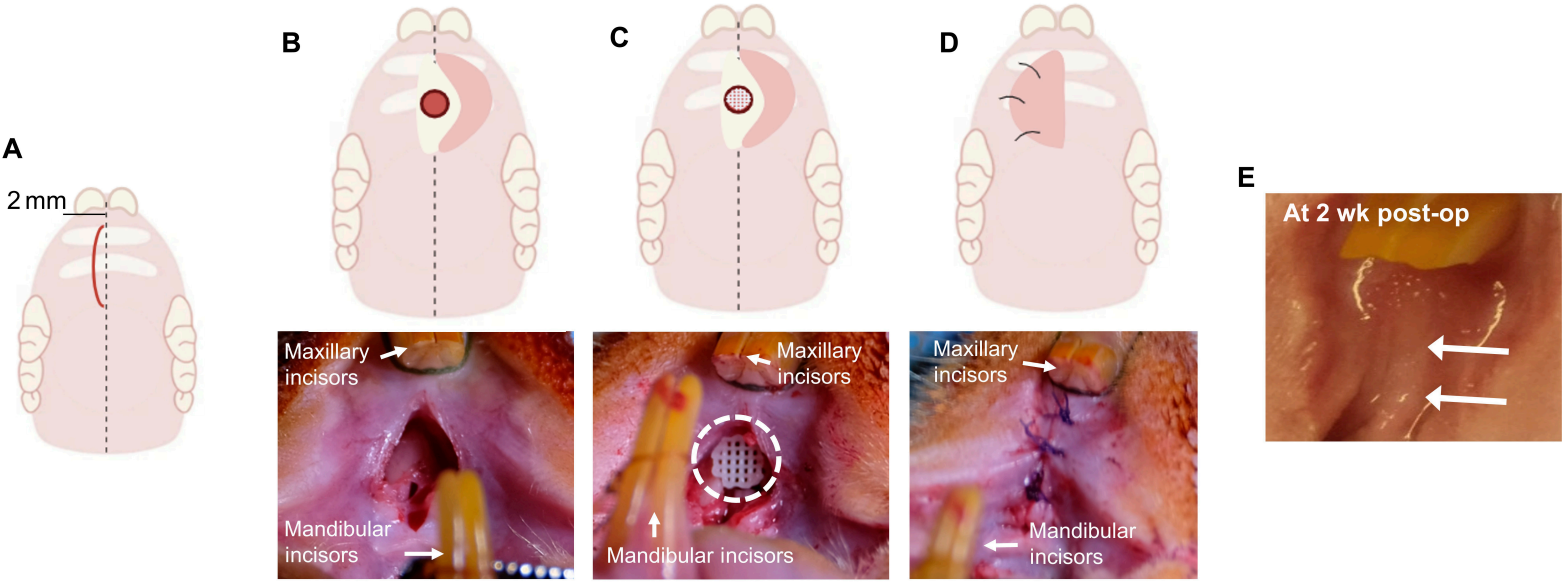
Surgical repair of rat palatal defects. A 2-mm incision was made caudal to the incisors (A), and flap was lifted to expose the surgical site (B). After creating a 3 × 0.5 mm bone defect, 3D-printed scaffolds were implanted (C), followed by tissue closure (D). Soft tissue healing was observed by 2 weeks post-op (E).

The in vivo efficacy of 3D-printed ICA/PCL scaffolds was tested in a rat premaxillary defect model with a full-thickness defect connecting the oral and nasal cavities to demonstrate adequate bone formation for the palatal base under the influence of oral habits and the microbiome [26]. This model allows normal feeding as the defect does not interfere with the normal dentition of the animal. Following an Institutional Animal Care and Use Committee-approved protocol, skeletally mature Sprague Dawley rats were used for the study (*n* = 14; 10- to 12-week-old males, weighing 400 to 500 g; Charles River, MA, USA). Under general anesthesia delivered by a ketamine/xylazine cocktail (60 mg/kg ketamine and 10 mg/kg xylazine) intraperitoneally, the rats were secured in a supine position with the jaw propped open to enable access to the palate. All oral tissues were disinfected with betadine. Lidocaine with 1:200,000 epinephrine (2 mg/kg lidocaine and 5 mg/kg epinephrine) was delivered as local anesthesia at the premaxillary soft tissue surgical site. The underlying premaxilla was exposed after a 2-cm midsagittal incision through the palatal gingival epithelium with a Bard-Parker #10 scalpel (Fig. 3A and B). A 3-mm carbide jobber drill with abundant saline was used to create a round defect where the scaffold was implanted at the defect site (Fig. 3C). The gingival flap was repositioned before achieving primary closure with a nonabsorbable nylon surgical monofilament suture (Fig. 3D). Intraoral sutures were removed at 2 weeks, after soft tissue healing and closure were achieved (Fig. 3E). Animals were randomly divided into 3 groups: (a) defect alone (defect covered with no scaffold; *n* = 4), (b) PCL-only scaffold (*n* = 5), and (c) ICA/PCL scaffold (*n* = 5). Based on the in vitro dosing study, 0.3 wt% ICA was selected for the in vivo study.

### Micro-CT analysis

The premaxillary defect site at 0-, 2-, 4-, 6-, and 8-week time points were scanned using micro-computed tomography (micro-CT; PerkinElmer Quantum FX micro-CT imaging system, Waltham, MA, USA). The region of interest was selected at the surgical defect-implantation site. The animals received ~400 mGy per scan for 4 min with a field of view of 40 mm. From the micro-CT images, bone volume (BV)/total volume (TV) was quantitatively compared between groups.

### In vivo histological evaluation

The skull samples harvested at 8 weeks post-op were decalcified with 10% ethylenediaminetetraacetic acid, embedded in paraffin, and then sliced along the sagittal plane in the defect-repair area. The 5-μm-thick sections were stained with hematoxylin and eosin and Picrosirius Red per our established protocols [13,22].

### Statistical analysis

All quantitative data from all groups were analyzed by one-way analysis of variance with Bonferroni multiple comparison tests at an *α* level of 0.05 after confirmation of normal data distribution using PRISM ver. 11 (GraphPad Software, San Diego, CA).

## Results

### Characterization of ICA-releasing 3D-printed PCL scaffolds

**Fig. 4.**
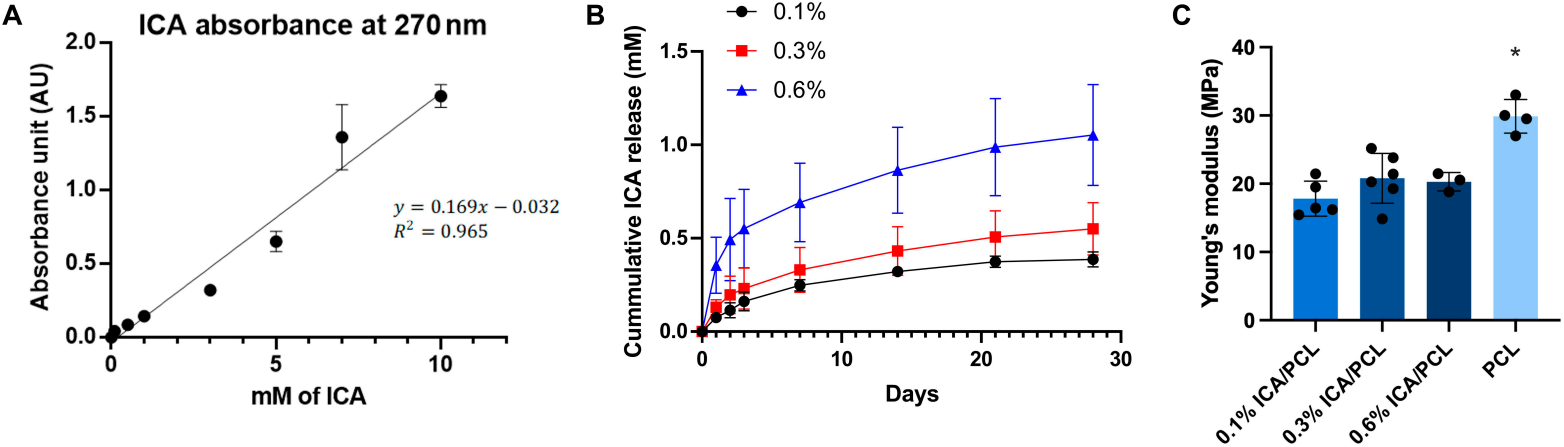
Standard curve of ICA (0 to 10 mM) at 270-nm absorbance (A). In vitro release kinetics of ICA at different loading doses (0.1% to 0.6%) (B). Young’s modulus of ICA-loaded PCL scaffolds (C) (*n* = 5 per group; *P < 0.001 compared to all other groups).

A standard curve of ICA at 270-nm absorbance (Fig. 4A) was used to calculate the released ICA. In vitro release kinetics showed sustained ICA release up to 28 d, with higher loading doses leading to higher concentrations of ICA release (Fig. 4B). The initial accelerated release phase is demonstrated by day 7, and the release plateaued after until day 21. On day 28, 0.1% ICA/PCL released 0.39 ± 0.04 mM, 0.3% ICA/PCL released 0.55 ± 0.14 mM, and 0.6% ICA/PCL released 1.05 ± 0.27 mM ICA. The Young’s moduli of ICA-loaded PCL scaffolds were approximately 65% to 70% of that of PCL scaffolds, with no statistically significant differences between 0.1%, 0.3%, and 0.6% ICA (Fig. 4C) (*P* < 0.001; *n* = 5 per group).

### In vitro osteogenic differentiation

**Fig. 5.**
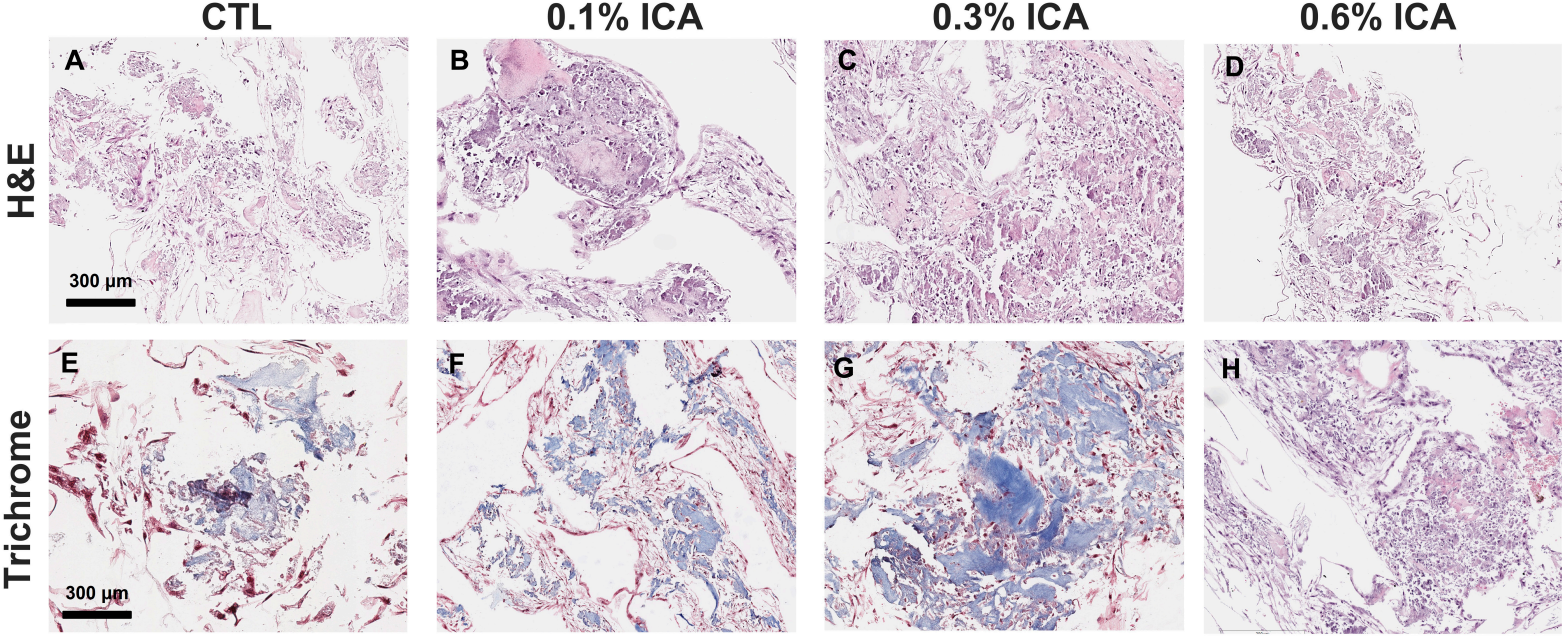
Histological analysis of mesenchymal stem cell (MSC)-seeded ICA/PCL scaffolds after 4-week culture in osteogenic differentiation media. Hematoxylin and eosin (H&E) (A to D) and trichrome (E to H) staining show new tissue formation in all groups, with collagenous matrix deposition notable in the 0.1% and 0.3% ICA groups (F and G) (representative images of *n* > 10 sections per sample).

**Fig. 6.**
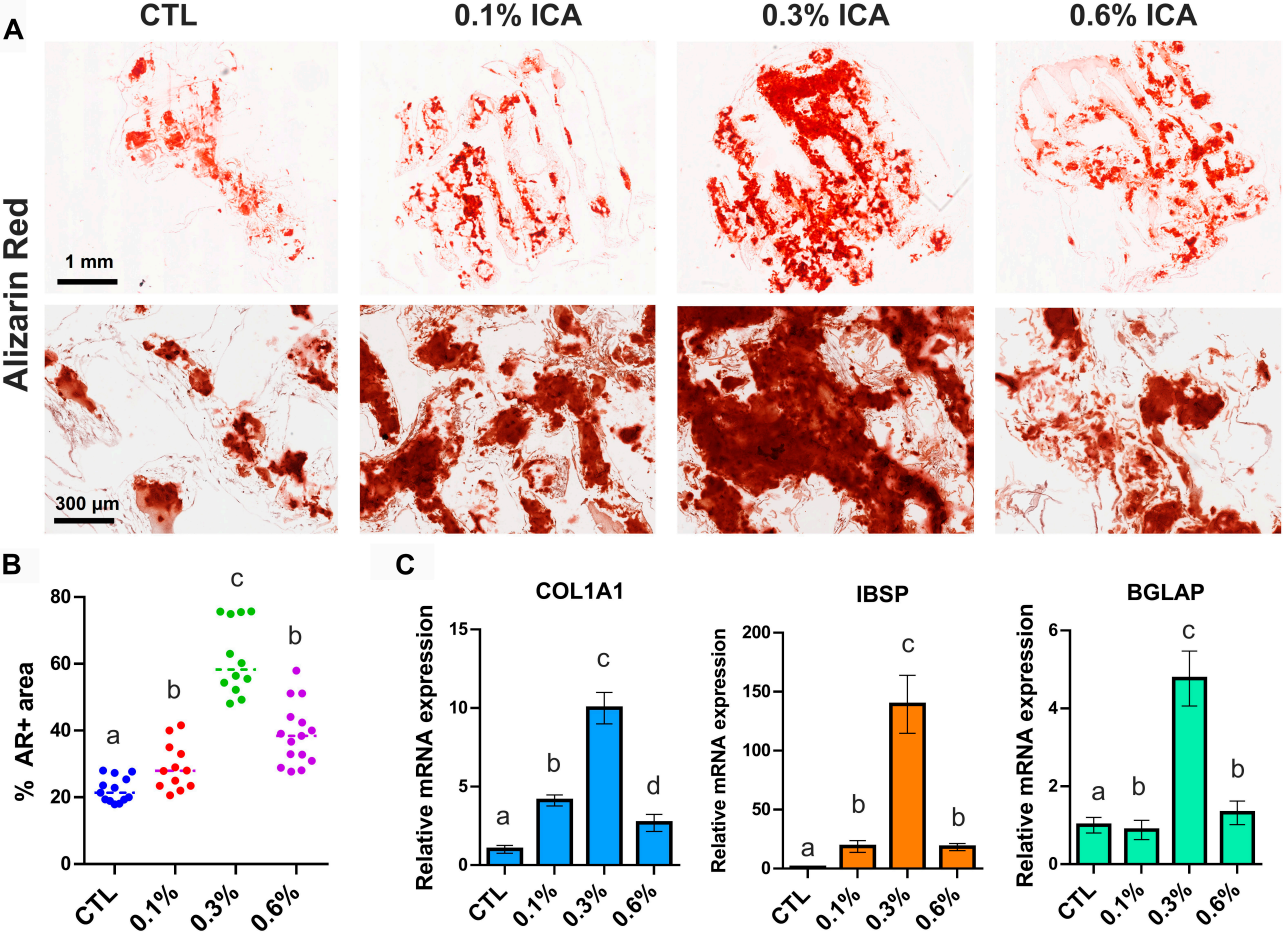
Osteogenic differentiation was promoted in ICA-loaded PCL scaffolds. Alizarin Red (AR) staining show increased calcium depositions in ICA-loaded PCL scaffolds, the most abundantly with 0.3% ICA (A). Quantitatively, the AR+ area was significantly higher in 0.3% ICA than in all other groups (B) (*n* = 12 to 15 per group; different letters indicate significant difference at *P* < 0001). Consistently, COL1A1, integrin-binding sialoprotein (IBSP), and bone gamma-carboxyglutamate protein (BGLAP) expressions were significantly higher with 0.3% ICA (C) (*n* = 5 per sample; different letters indicate significant difference at *P* < 0001). CTL, control; mRNA, messenger RNA.

After 4-week culture in osteogenic differentiation media, all MSC-seeded scaffold constructs showed de novo tissue formation (Fig. 5A to D). Trichrome-stained sections revealed notable collagen deposition in 0.1% ICA/PCL and 0.3% ICA/PCL scaffolds (Fig. 5F and G), as compared to control and 0.6% ICA/PCL scaffolds (Fig. 5E and H). Among all groups, 0.3% ICA/PCL appeared to show the most abundant collagenous matrix formation (Fig. 5G). Consistently, 0.3% ICA/PCL showed the most robust mineralized matrix formation per AR staining, compared to all other groups (Fig. 6A). Quantitatively, the AR-positive area was significantly bigger in 0.3% ICA/PCL than in all other groups (Fig. 6B) (*P* < 0.001; *n* = 11 to 15 per group). In addition, the 0.3% ICA/PCL group resulted in the highest expressions of osteogenic markers, including COL1A1, IBSP, and BGLAP, among all of the tested groups (Fig. 6C) (*n* = 5 per group; *P* < 0.001). We selected 0.3% ICA/PCL scaffolds for our in vivo experiments based on these findings.

### In vivo alveolar cleft bone healing

**Fig. 7.**
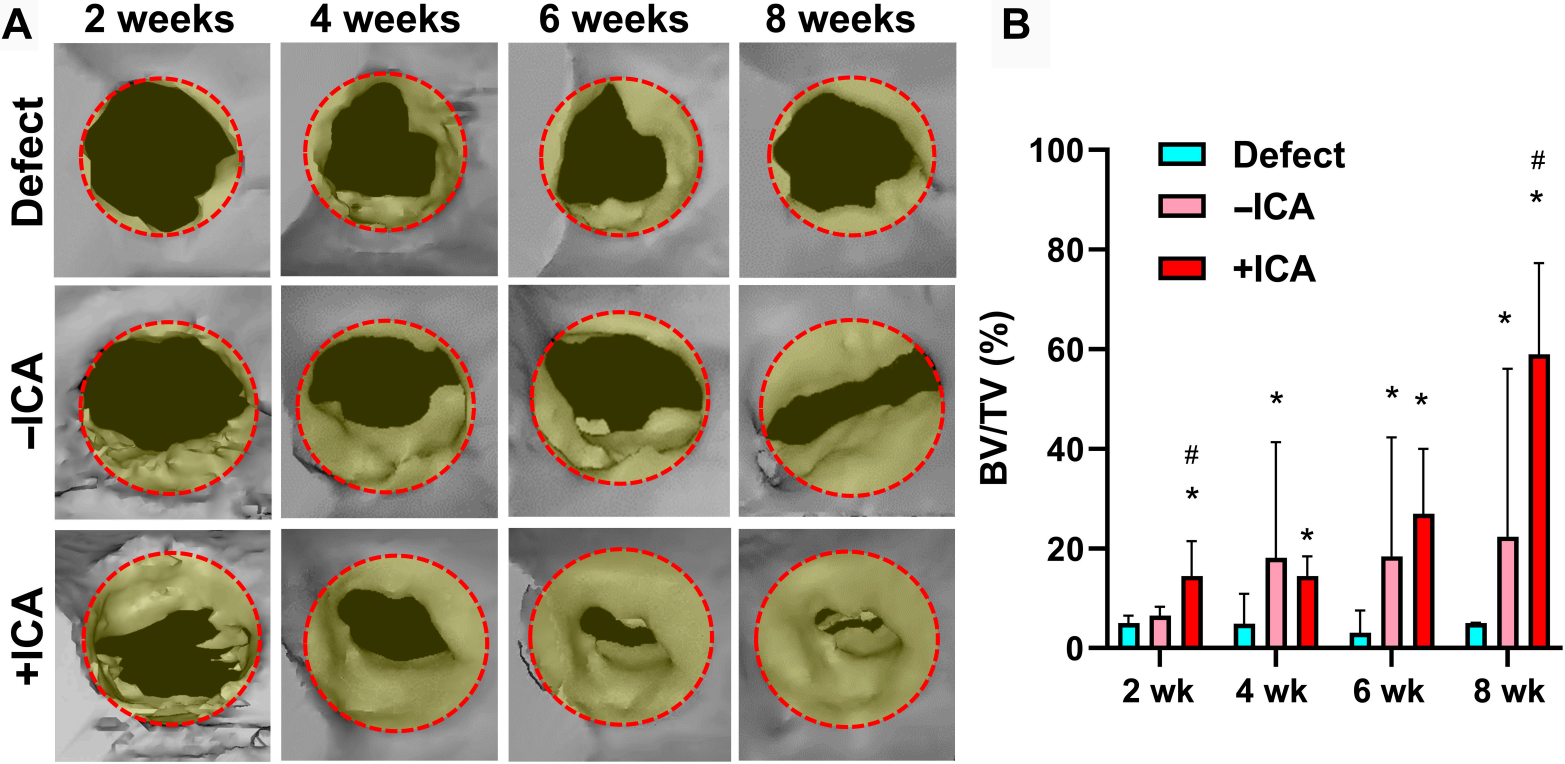
Micro-computed tomography (micro-CT) analysis of alveolar bone healing in vivo. For 2 to 8 weeks post-op, micro-CT images show new bone formation in the defect sites, with accelerated bone healing with scaffolds + ICA (A). Quantitatively, bone volume (BV)/total volume (TV) was significantly higher with scaffolds + ICA as compared to those with scaffolds − ICA and the defect-only control (B) (*n* = 5 per group; *P < 0.001 compared to defect; ^#^*P* < 0.001 compared to −ICA).

**Fig. 8.**
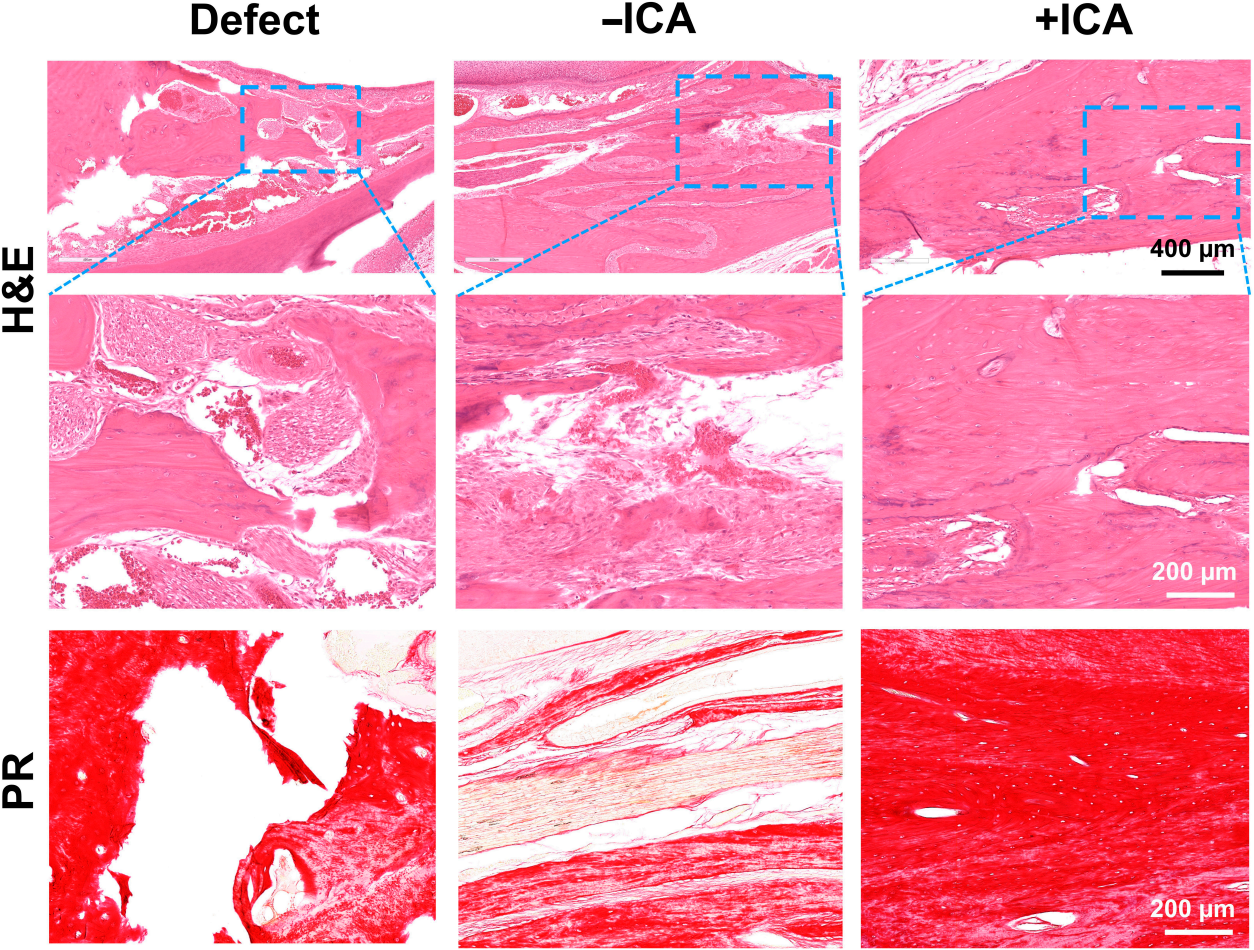
Histology of in vivo samples harvested at 8 weeks post-op. H&E and Picrosirius Red (PR) staining suggest unclosed defects in the control group. Scaffold − ICA resulted in immature tissue healing with soft tissue infiltration, and scaffolds + ICA show a closed gap with a mature, woven bone structure. Images were taken at the central zone of the defects, and no sign of the remaining scaffold was observed.

Despite an ~10% reduction in body weight for 3 to 5 d postsurgery, all animals returned to their original weight and presented regular feeding until sacrifice, maintaining a healthy body weight. By 2 to 8 weeks, micro-CT analysis showed unclosed bone defect without treatment (Fig. 7A). In contrast, the bone defect implanted with scaffolds + ICA presented accelerated bone healing over the period, resulting in nearly closed gaps by 8 weeks (Fig. 7A). Scaffold − ICA showed better bone healing but gaps remained (Fig. 7A). Quantitatively, BV/TV was significantly higher in the +ICA group than in the −ICA group at 2 and 8 weeks (Fig. 7B) (*n* = 5 per group; *P* < 0.001). At all time points, +ICA and −ICA showed significantly higher BV/TV compared to the untreated control (Fig. 7B) (*P* < 0.001). Consistently, histology showed unclosed defects in the control group by 8 weeks. In contrast, scaffold + ICA resulted in closed defects with matured woven bone (Fig. 8). Scaffolds without ICA showed tissue healing with soft tissue and immature mineralization (Fig. 8).

## Discussion

Our findings suggest the promising potential of cell-free, ICA-releasing 3D-printed scaffolds for facilitating alveolar cleft bone reconstruction. Our study determined an effective dose and the release kinetics of ICA for promoting osteogenic differentiation of MSCs, leading to accelerated bone regeneration in the rat premaxillary defect model. Interestingly, 0.3% ICA was more effective than 0.1% and 0.6%, suggesting a dose-dependent functionality of ICA when delivered via a 3D-printed PCL scaffold, despite the lack of clear understanding of its mechanism. Given no need for a growth factor or cell transplantation, our ICA-releasing scaffolds may represent a highly translational graft to improve the clinical outcome of cleft palate reconstruction surgery. Although growth factors (e.g., bone morphogenetic proteins) exhibit osteogenic potential, the development cost of a clinically applicable scaffold delivered with a growth factor is of a higher magnitude of order than that of scaffolds with a small molecule [27]. Moreover, ICA is a plant-derived molecule, thus significantly simplifying the regulatory requirements for its clinical translation [28]. Importantly, our ICA-loaded scaffold can be fabricated in hours in a patient-specific design with the benefits of 3D printing technology.

Various types of alveolar bone healing animal models have been used to test tissue-engineered bone grafts for cleft palate reconstruction [9–11,29,30]. One of the most used models is the rat calvaria defect model [17,31–33]. Although widely used, the calvaria defect model cannot capture the oral microbiome and mechanical loading during feeding and bruxism. Several previous studies used a unilateral alveolar defect, also referred to as gingivoperiosteoplasty [29,30,34]. However, gingivoperiosteoplasty is limited in testing the efficacy of bioengineered grafts in closing the oronasal cavity. Our model with a full-thickness defect connecting the oral and nasal cavities is adequate in testing bone formation for the palatal base while exposed to the microbiome. This model also allows regular feeding as the defect does not interfere with the animal’s normal dentition. Although all existing animal models, including ours, cannot fully replicate cleft palates in human patients, the significantly facilitated alveolar bone regeneration in a rat cleft palate model may suggest the clinical potential of the ICA/PCL scaffolds.

The mechanical properties of scaffolds are one of the most important factors to be considered for bone reconstruction [35]. During deglutition, the alveolar bone and hard palate are subjected to negative pressure [36–38] as the contraction of intraoral muscles and the anterior tongue generates a negative pressure wave as the tongue exerts an upward force against the palate. This pressure wave compresses the elastic oral walls, creating a closed and restricted environment [36]. As healthy individuals swallow approximately 585 times per day [39], scaffold materials should possess mechanical properties sufficient to provide necessary mechanical integrity during bone healing [40]. Although loading ICA into PCL scaffolds somewhat reduced the mechanical properties, our ICA/PCL scaffolds showed no sign of structural breakdown for up to 8 weeks of implantation, as being entirely replaced by newly forming tissues.

Despite the promising in vivo outcomes, this study has several limitations. First, we do not have quantitative data on the in vivo degradation rate of the ICA/PCL scaffolds. Biodegradable polymeric scaffolds may degrade at different rates depending on various factors, including but not limited to (a) porosity and surface area (determined by the size of strands and interstrand distance in the 3D-printed scaffold), (b) the molecular weight of materials, and (c) mechanical and biochemical stimulants in local environments [41]. Thus, future studies will establish comprehensive degradation data for alveolar bone cleft regeneration, which are required for determining the designing parameters of scaffolds for large animals or human patients. The second limitation is the lack of mechanistic study. Previous studies suggested several signaling pathways involved in ICA’s osteogenic function, including Wnt, mitogen-activated protein kinase, and cyclic adenosine monophosphate [14,15]. In addition, ICA may have immunomodulatory functions associated with bone regeneration [42]. However, we do not have a clear understanding of the underlying mechanism for alveolar bone regeneration via ICA/PCL scaffolds, potentially involved with an endogenous mesenchymal cell source and interaction with the immune system. Another limitation of this study is the short-term follow-up in vivo. A long-term follow-up is necessary to determine whether de novo bone can undergo continuous remodeling, thus preventing repetitive surgeries as patients grow. Finally, these scaffolds will require study in large animals.

In conclusion, our study suggests the potential of the ICA-releasing 3D-printed PCL scaffold for accelerated bone healing in alveolar cleft reconstruction. As a cell-free scaffold activated by a plant-derived small molecule, the ICA-releasing scaffold may have fewer translational barriers as compared to scaffolds combined with growth factors and/or cells.

## Data Availability

The data that support the findings of this study are available from the corresponding author, C.H.L., upon reasonable request.
